# Development and validation of a highly accurate multigene gene expression biomarker to predict chemotherapy response in primary triple-negative breast cancer

**DOI:** 10.1007/s10549-026-07950-4

**Published:** 2026-03-26

**Authors:** Soukaina Amniouel, Mohsin Saleet Jafri

**Affiliations:** 1 School of Systems Biology, George Mason University, Fairfax, VA 22030 USA; 2 Center for Biomedical Engineering and Technology, University of Maryland School of Medicine, Baltimore, MD 21201 USA

**Keywords:** TNBC, Cancer, Chemotherapy, Machine learning, Gene expression

## Abstract

**Purpose:**

Triple-negative breast cancer (TNBC) is an aggressive subtype lacking estrogen and progesterone receptors and HER2 amplification. Representing 10–15% of breast cancer cases, TNBC disproportionately affects Black and pre-menopausal women and is associated with poorer outcomes. With chemotherapy as the primary systemic treatment option, achieving a pathological complete response (pCR) to neoadjuvant chemotherapy (NAC) is a key prognostic factor. However, TNBC biological heterogeneity complicates treatment response prediction. This study aimed to identify transcriptomic biomarkers predictive of NAC response in TNBC patients and evaluate machine-learning models for response classification.

**Methods:**

We performed transcriptomic profiling on tumors from 234 TNBC patients, divided into training 138 pCR,72 residual disease (RD) and test 9 pCR, 15 RD cohorts. Feature selection was conducted using LASSO regression and Boruta algorithms to identify robust biomarkers. Random forest and support vector machine (SVM) models were trained on the selected and evaluated on the independent test set.

**Results:**

Feature selection identified 21 overlapping biomarkers, including EPHB3, ATP5MJ, USP1, RANBP9, SLC11A2, S100P, PPP1R1A, ZIC1, NDRG2, SMARCA2, H2BC7, STK24, HBB, VPS45, H1, VEGFA, NFIB, ITGA6, RPRD1A, PRKD3, and ENSA, several of which have been implicated in TNBC progression and treatment resistance. In the test set, predictive performance was strong, with area under the curve (AUC) values of 91% for random forest and 89% for SVM.

**Conclusion:**

Transcriptomic profiling combined with machine learning provides a promising approach for predicting NAC response in TNBC. The identified biomarkers may inform precision treatment strategies and improve clinical outcomes in this high-risk patient population.

**Supplementary Information:**

The online version contains supplementary material available at 10.1007/s10549-026-07950-4.

## Introduction

Triple-negative breast cancer accounts for roughly 15–20% of all breast cancer cases and is defined by the absence of estrogen receptors (ER), progesterone receptors (PR), and human epidermal growth factor receptor 2[1]. Clinically, TNBC is highly aggressive: tumors are often high-grade and proliferative, and due to a lack of targeted therapies, this subtype carries the poorest prognosis among breast cancers[1].

Neoadjuvant chemotherapy (NAC) is a mainstay treatment for early-stage TNBC in an effort to improve surgical outcomes and eliminate micro-metastases. Standard anthracycline-and taxeme-based NAC regimens (e.g., TFAC or TFEC) yield a pathological complete response in only a subset of patients (approximately 30–65%) [2]. Achieving pCR is strongly associated with favorable long-term outcomes, whereas patients who do not reach pCR (i.e., have RD) experience substantially higher relapse rates and poorer survival [2, 3]. The complex biology of TNBC involves dysregulated signaling pathways such as NF-κB, PI3K/AKT/mTOR, JAK/STAT, and EGFR, contributing to its resistance to chemotherapy [4, 5]. This variability in response highlights the urgent need for predictive biomarkers to predict which TNBC patients will respond to NAC [6, 7]. Identifying robust markers of chemosensitivity or resistance could enable clinicians to personalize treatment, improving outcomes for responders and sparing non-responders unnecessary toxicity.

By profiling tumors at the genome, transcriptome, and proteome levels, researchers can uncover molecular signatures associated with drug response, and ML algorithms can integrate these complex data to make predictive models [8–10]. Early studies demonstrate the feasibility of this approach: multi-parameter models that combine tumor-intrinsic features and immune biomarkers, such as gene expression profiles or tumor-infiltrating lymphocyte levels, have shown improved ability to predict NAC outcomes in TNBC [6, 7, 11]. These findings suggest that incorporating diverse biological markers via bioinformatics and ML can enhance response prediction, moving toward more personalized therapy.

In the present study, we apply integrative bioinformatics and ML techniques to identify predictive biomarkers of response to anthracycline-taxane-based NAC (e.g., TFAC and TFEC) in TNBC. Our goal is to develop a predictive framework that can stratify TNBC patients by likely chemo-responsiveness, ultimately paving the way for more individualized and effective treatment strategies in this aggressive breast cancer subtype.

## Material and methods

The present study follows an established machine-learning pipeline for data collection, preprocessing, feature selection, classification modeling, and biological interpretation, previously described in detail and applied in our prior solid tumor studies for predicting therapeutic response [9, 10].

### Data collection

We sourced gene expression data from the Gene Expression Omnibus (GEO) database using the GEOquery R package. Keywords used included “Triple-Negative-Breast-Cancer,” “Chemotherapy,” “Expression profiling by array,” and “Homo-sapiens.” We focused on anthracycline-taxed-based NAC regimens, specifically TFAC (Taxol, Fluorouracil, Anthracycline and cyclophosphamide) and TFEC (Taxol, Fluorouracil, Epirubicin and cyclophosphamide). Three GEO datasets (i.e., GSE20194, GSE20271, and GSE25065) were selected, all from the Affymetrix Human Genome U133A platform (Table 1).

**Table 1:** GEO accession numbers, platforms, sample counts (pCR, RD, total), and associated references

GEO accession	Platform	Number of samples	Title/description
GSE20194	GPL96-HG-U133A	278	MAQC-II Project: human breast cancer (BR) data set
GSE20271	GPL96-HG-U133A	178	Expression data from breast cancer FNA biopsies from patients
GSE25065	GPL96-HG-U133A	198	Validation cohort for genomic predictor of response and survival following neoadjuvant taxane-anthracycline chemotherapy in breast cancer

^1^GPL96 = Affymetrix GeneChip Human Genome U133 Array (HG-U133A)

### Data preprocessing

CEL files from three independent datasets (*n* = 278, 178, and 198 samples) were initially collected. Samples were filtered to retain only TNBC cases with available chemotherapy response information. Samples not related to TNBC or with missing clinical or response data were excluded. The remaining CEL files were merged and normalized using the GC Robust Multi-array Average (GCRMA) method. Probe IDs were mapped to gene symbols via the R package “org.Hs.eg.db.” Prior to differential expression analysis, the merged dataset comprising 234 samples was randomly divided into a training cohort (*n* = 210, 90%) and an independent validation cohort (*n* = 24, 10%) using stratified sampling implemented in the caret package to preserve class proportions. A fixed random seed was used to ensure reproducibility.

After standard data preprocessing and filtering, a total of 6323 genes were retained for downstream analysis. All subsequent feature selection including differentially expressed analysis was performed using the training cohort only to identify genes associated with treatment response. Gene expression differences between response groups were assessed using the limma package in R, which fits linear models to expression data and applies empirical Bayes moderation. Genes with adjusted p-value < 0.1 and |log2 fold change|> 1 were considered differentially expressed. Differential expression results were visualized using volcano plots generated with the EnhancedVolcano package in R [12–14].

### Functional enrichment analysis

Functional enrichment analysis, including Gene Ontology (GO), and Kyoto Encyclopedia of Genes and Genomes (KEGG) pathway analyses were performed using “clusterProfiler” (*p* < 0.05) in R based on DEGs identified in the training cohort [15]. Ingenuity Pathway Analysis (IPA) software was used to explore biological interactions and identify significant regulatory networks[16].

### Feature selection

Feature selection was performed on the training-set differentially expressed genes using Least Absolute Shrinkage and Selection Operator (LASSO) and Boruta. LASSO was employed to perform regularization-based feature selection by shrinking less informative coefficients toward zero, thereby reducing model complexity and mitigating overfitting, whereas Boruta, a tree-based wrapper method, was used to identify features with statistically significant importance. To enhance model robustness and generalizability, only genes selected by both methods were retrained for downstream machine-learning modeling [17–20].

For LASSO, the regularization parameter (λ) was selected using tenfold cross validation. To assess the robustness of selected features, we performed resampling-based feature stability analysis. Specifically, the training set was repeatedly subsampled (80% of samples per run, stratified by drug response) for B = 100 iterations. In each iteration, feature selection was re-applied from scratch, and features with non-zero coefficients (LASSO) or classified as “Confirmed” (Boruta) were recorded. For each feature, a stability score was computed as the proportion of resampling runs in which it was selected. Features selected in at least 70% of runs were considered stable and used for downstream modeling and biological interpretation.

### Machine-learning models

Classification models were trained using the features identified through stability-based feature selection. Random forest (RF) and Support Vector Machine (SVM) were implemented using the training set only via the Random Forest and e1071 R packages, respectively. Model hyperparameters were optimized using tenfold cross validation within the training set. For RF, the number of variables randomly sampled at each split (mtry) was tuned, while for SVM with a radial basis function kernel, the cost and kernel parameters were optimized. Model performance was evaluated using the area under the curve (AUC), accuracy, sensitivity, and specificity. After hyperparameter tuning, the final models were trained on the full training set and evaluated once on the held-out validation set that was not used for feature selection, model training, or hyperparameter tuning.

## Results

### Data acquisition and primary analysis

The final dataset included 234 patients, of whom 81 achieved pCR and 153 had residual disease. The training and validation cohorts consisted of 210 and 24 patients, respectively, with similar outcome contributions. 

### Identification of PCR-TNBC-related DEGs.

**Fig. 1:**
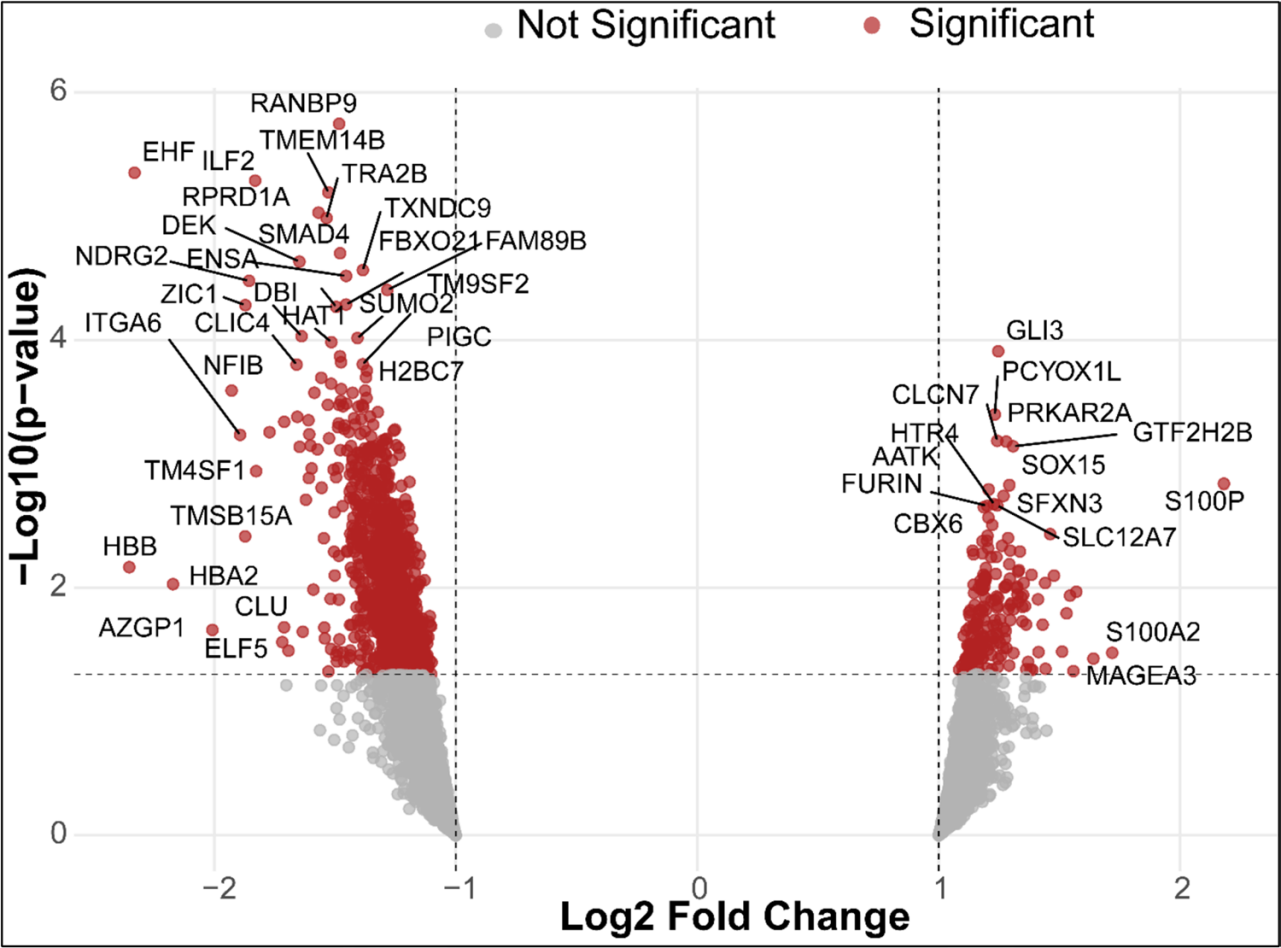
Volcano plot of differential expression analysis. The x-axis represents log2 fold change, and the y-axis shows the statistical significance (-log10(p-value)). Thresholds applied are log2FC ≥ 1 and adjusted *p*-value < 0.05 with the genes highlighted in red

- Differential expression analysis performed in the training cohort identified 441 out of 6323 genes significantly associated with treatment response that were found to be upregulated and 408 were downregulated in TNBC patients with pCR. Several of the top differentially expressed genes include *EHF, ILF2, TMEM14B, RANBP9, RPRD1A, TRA2B, SMAD4, DEK, NRDG2, ENSA,* and others (Fig. 1**; **Table 2). The overexpression of these genes has been previously associated with cancer progression in TNBC.

**Table 2:** Top 10 upregulated and downregulated genes from differential expression analysis

Upregulated			Downregulated		
Genes	Log2FC	Adj. P-Value	Genes	Log2FC	Adj. P-Value
GLI3	1.24	0.04	EHF	− 2.33	0.01
GTF2H2B	1.30	0.05	ILF2	− 1.83	0.01
PRKAR2A	1.27	0.05	TMEM14B	− 1.52	0.01
CLCN7	1.24	0.05	RANBP9	− 1.48	0.01
PCYOX1L	1.23	0.05	RPRD1A	− 1.56	0.01
S100P	2.18	0.06	TRA2B	− 1.53	0.01
SOX15	1.29	0.06	SMAD4	− 1.47	0.01
AATK	1.20	0.06	DEK	− 1.64	0.01
SFXN3	1.26	0.07	NRDG2	− 1.85	0.01
SLC12A7	1.24	0.07	ENSA	− 1.45	0.01

For each gene, metrics such as Log_2_ Fold Change (Log_2_FC) and adjusted p-value (Adj. *P*-Value) are provided, demonstrating significant change in expression between pCR and RD

### Identification of functional and pathways enrichment in PCR TNBC

**Fig. 2:**
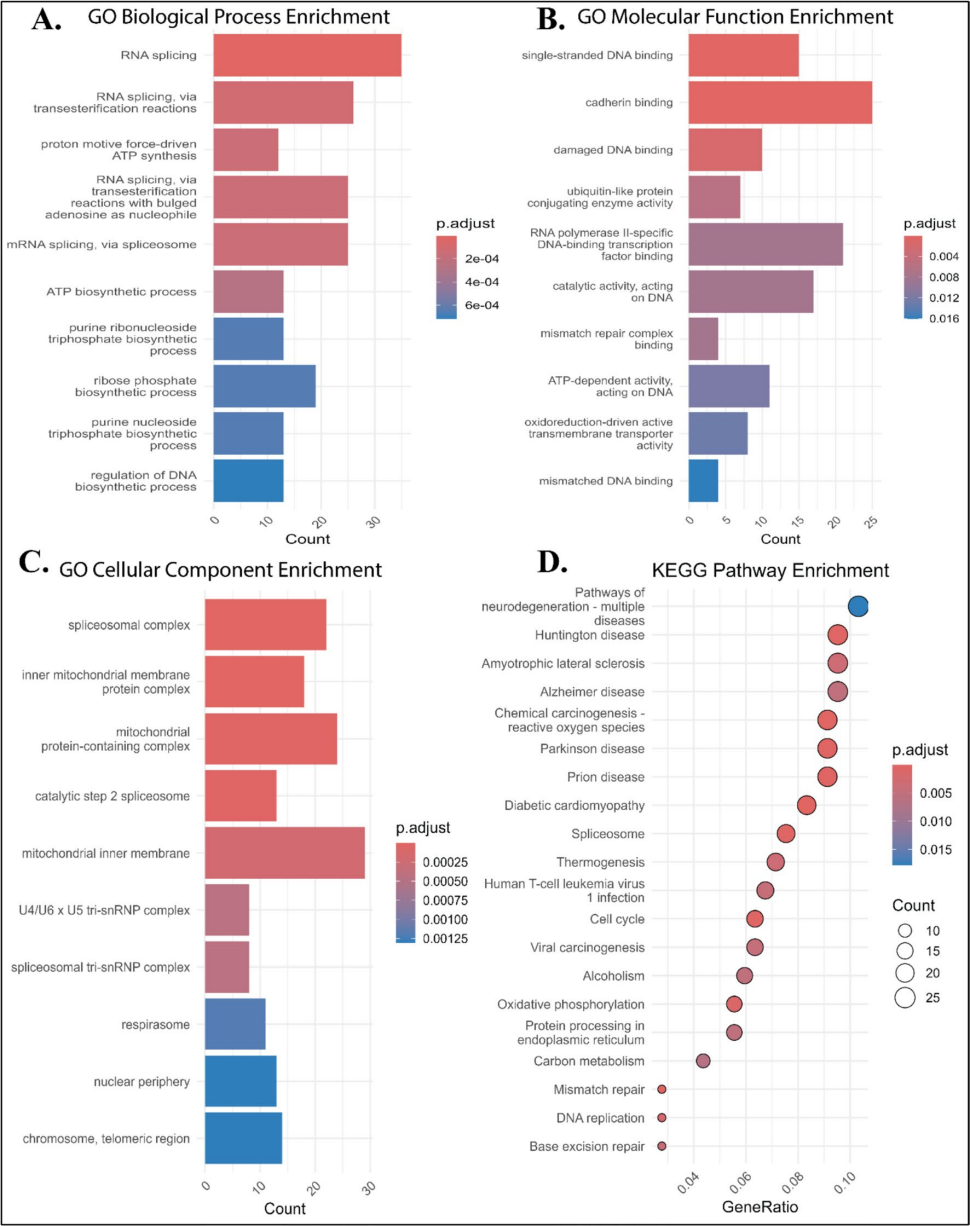
Functional enrichment analysis (Gene Ontology and KEGG pathway). Enrichment analysis results across multiple categories: **A** Biological Processes (GO-BP), showing enriched biological processes; **B** Molecular function (GO-MF), highlighting key molecular activities; **C** Cellular Component (GO-CC), demonstrating the cellular localization of functions; and **D** KEGG pathway, summarizing pathway-level enrichment. The findings provide an overview of the functional roles as well as pathways associated with TNBC

The GO Biological Process (BP) analysis revealed enrichment in RNA splicing, proton motive force-driven ATP synthesis, ribonucleoside triphosphate biosynthetic process, regulation of DNA biosynthetic process, and energy derivation by oxidation of organic compounds (Fig. 2A). GO Molecular Function (MF) terms included single-stranded DNA binding, cadherin binding, damaged DNA binding, ubiquitin-like protein conjugation enzyme activity, and RNA polymerase II-specific DNA-binding transcription factor binding (Fig. 2B), while GO Cellular Component (CC) terms were enriched in spliceosomal complex, inner mitochondrial membrane protein complex, U4/U6 x U5 tri-snRNP complex, respirasome, nuclear periphery, and chromosome and telomeric region (Fig. 2C). KEGG pathway analysis revealed multiple relevant pathways among the top 20, such as amyotrophic lateral sclerosis, chemical carcinogenesis–reactive oxygen species, diabetic cardiomyopathy, spliceosome, thermogenesis, Human T-cell leukemia virus 1 infection, cell cycle, viral carcinogenesis, alcoholism, oxidative phosphorylation, protein processing in endoplasmic reticulum, mismatch repair, DNA replication, and base excision repair (Fig. 2D).

**Fig. 3:**
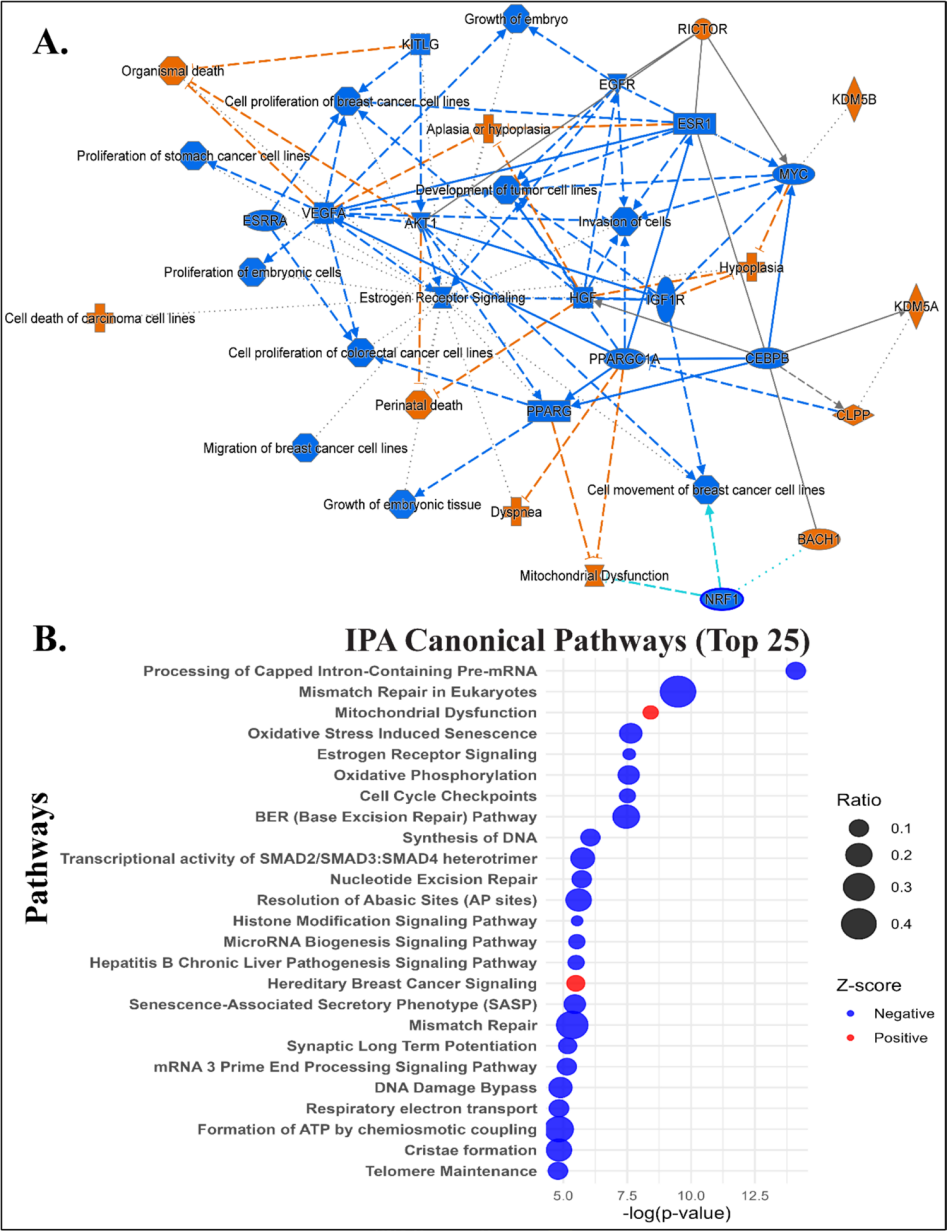
Graphical summary and canonical pathway from ingenuity pathway analysis (IPA). **A** The graphical summary plot provides an overview of the important biological processes, diseases, and molecular interactions derived from the IPA knowledge base. It demonstrates interconnected pathways and their predicted activation (orange) or inhibition (blue) states. The solid line arrow represents a direct connection, whereas the dashed arrow represents an indirect connection. **B** The canonical pathway bubble plot demonstrates the top 25 canonical pathways ranked by their significance (-log(p-value)) and Z-score. The bubble size reflects the ratio of pathway-associated genes identified in the dataset, while the color represents the Z-score directionality; red for activated pathways and blue for inhibited pathways. In addition, pathways are sorted for clarity, emphasizing the most relevant biological findings

Ingenuity pathway analysis (IPA) highlighted critical regulatory networks involving the DEGs (Fig. 3A). Notably, the suppression of estrogen receptor signaling was linked to increased organismal death and reduced cell proliferation, indicating its key role in TNBC progression. Also, genes such as AKT1, ESR1, HGF, IGF1R, MYC, VEGFA, and PPARG were associated with cell proliferation and the development of tumor cell lines, with decreased activity correlated with reduced proliferation in breast cancer cell lines, underlying their role in oncogenesis and metastasis. Canonical pathways via IPA (Fig. 3B) identified 218 significantly enriched pathways based on adjusted p-values and Z-scores, including processing of capped intron-containing pre-mRNA, cell-cycle checkpoints, class I MHC-mediated antigen processing and presentation, neutrophil extracellular trap signaling pathway, chromatin organization, oxidative phosphorylation, synthesis of DNA, nucleotide excision repair (NER), ribonucleotide reductase signaling, and histone modification signaling pathway. Figure 3B represents the top 25 canonical pathways associated with the DEGs (Table 3 (Table 4).

**Table 3:** Summary of key biological pathways and associated molecular targets identified from gene expression analysis in triple-negative breast cancer (TNBC)

Pathways	Genes identified from the study
oxidative phosphorylation	*ATP5F1A, ATP5MC2/3, ATP5PF, COX5B, COX7A2L, COX8A, NDUFA5, NDUFB2/3/5/6,* and* SDHC*
oxidative stress pathway	*CBX6, CDK4, CDKN2C, H2BC12, H2BC9, MAP4K4, MAPK1, MAPK9, RBBP7, SCMH1, TFDP1, TFDP2*
diabetic cardiomyopathy	*EHF, CLIC4, H2BC10, SLC11A2, SP3, RNF138, USP1, ACTL6A, CAPRIN1, CDV3, PDK3, NARF, ACSL3, ATP5F1A, HNRNPA1, WEE1, MPC2, IQSEC1, CHEK1, RPL24, RNF14*
base excision repair (BER)	*FEN1, HMGB1, MAPK1, MBD4, PCNA, RFC2, RFC5, RPA1, UNG*
nucleotide excision repair (NER)	*ACTL6A, COPS8, CUL4B, PCNA, POLR2G, RFC2, RFC5, RPA1, SUMO1, SUMO2, UBE2N*
Homologous recombination	*CHEK1, H2BC12, H2BC9, PCNA, RFC2, RFC5, RPA1, SUMO2, TOPBP1, UBE2N*
DNA synthesis	*ANAPC5, CDC16, FEN1, GMNN, MCM3, MCM5, PCNA, PSMC3, RFC2, RFC5, RPA1, UBE2E1*
Cell Cycle	*ANAPC5, BUB3, CDC16, CHEK1, DYNC1I2, H2BC12, H2BC9, MAPRE1, MCM3, MCM5, PPP1CC, PPP2R5C, PSMC3, RFC2, RFC5, RPA1, TOPBP1, UBE2E1, UBE2N, WEE1*
Circadian rhythm	*ATF2, CREB1, CRY1, PER2, PPP1CB, PPP1CC, TBL1XR1*
Estrogen receptor negative	*ATF2, ATP5F1A, ATP5PF, CCNC, CREB1, EIF2B1, HSP90AB1, MAPK1, MED21, MED4, NDUFA5, NDUFB2/3/5/6, PCNA, PLCH1, PPP1CB, PRKAR1A/2A, PRKD3, RAP2A, SDHC, TBL1XR1, VEGFA*

**Table 4:** Performance metrics of machine-learning models including random forest and support vector machine (SVM)

Model		Accuracy	95% CI	Sensitivity	Specificity	AUC
Validation	*Random forest*	0.875	(0.6764, 0.9734)	0.933	0.777	0.881
	*Support vector machine*	0.875	(0.6764, 0.9734)	0.933	0.777	0.837

Metrics include accuracy, 95% CI sensitivity, specificity, and AUC

### Construction of TNBC related gene signature using feature selection

Feature selection was performed using LASSO regularization and the Boruta algorithm on the training set. Because feature selection may vary depending on the specific data partition, we assessed feature robustness using resampling-based stability analysis. Across B = 100 resampling runs, the number of features selected per run varied, indicating sensitivity to data perturbation. However, a subset of features was consistently selected across a large proportion of runs.

**Fig. 4:**
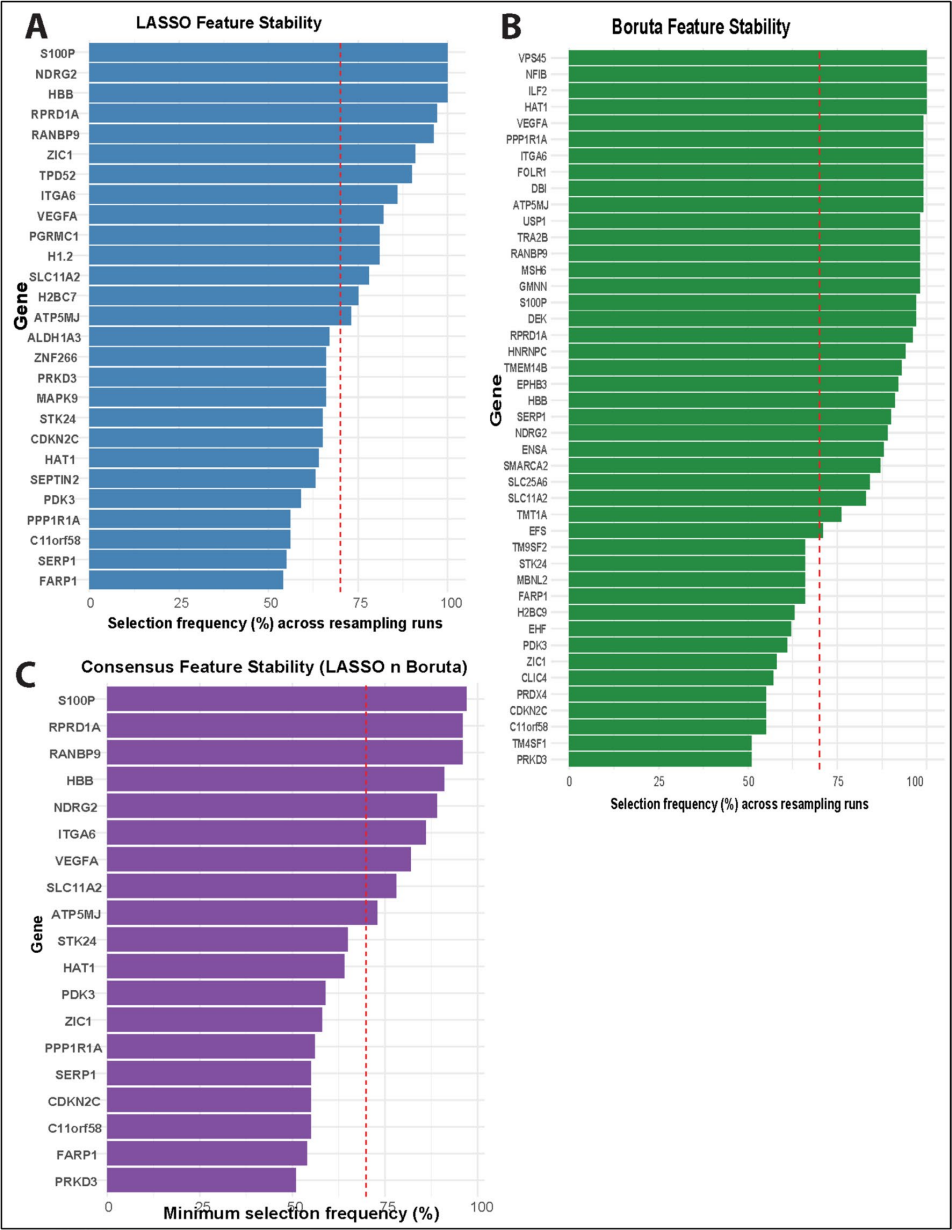
Stability-based feature selection using random forest and support vector machine approaches. Feature stability was assessed using repeated resampling of the training set (B = 100). **A** shows the frequency of features identified by Random Forest-based feature selection, **B** shows feature stability based on Support Vector Machine-based feature selection, and **C** shows the consensus features selected by both methods. Bars indicate the proportion of resampling runs in which each feature was selected. The dashed line denotes the stability threshold used to define robust features

For LASSO-based selection, 14 features were selected in at least 70% of resampling runs (Fig. 4B). Similarly, Boruta-based stability analysis identified 30 features that were confirmed in ≥ 70% of runs (Fig. 4C). These results suggest that only a limited subset of features exhibits robust associations with drug response.

To further prioritize robust predictors, we identified consensus features that were consistently selected by both LASSO- and Boruta-based stability analyses. This intersection yielded 9 features (Fig. 4D), representing genes that were robust to both linear (LASSO) and nonlinear (Boruta) feature selection approaches. For consensus features, the direction of LASSO coefficients was largely consistent across resampling runs, suggesting reproducible positive or negative associations with drug resistance. These consensus features were therefore used as inputs for the following machine-learning models for downstream biological interpretation. Among the consensus features were genes previously implicated in drug resistance.

### Machine learning model performance comparisons

RF and SVM classifiers were trained using the consensus feature set derived from stability-based feature selection and evaluated on an independent held-out validation set. Model performance was assessed using the area under the receiver operating characteristic curve (ROC AUC), sensitivity, and specificity.

**Fig. 5:**
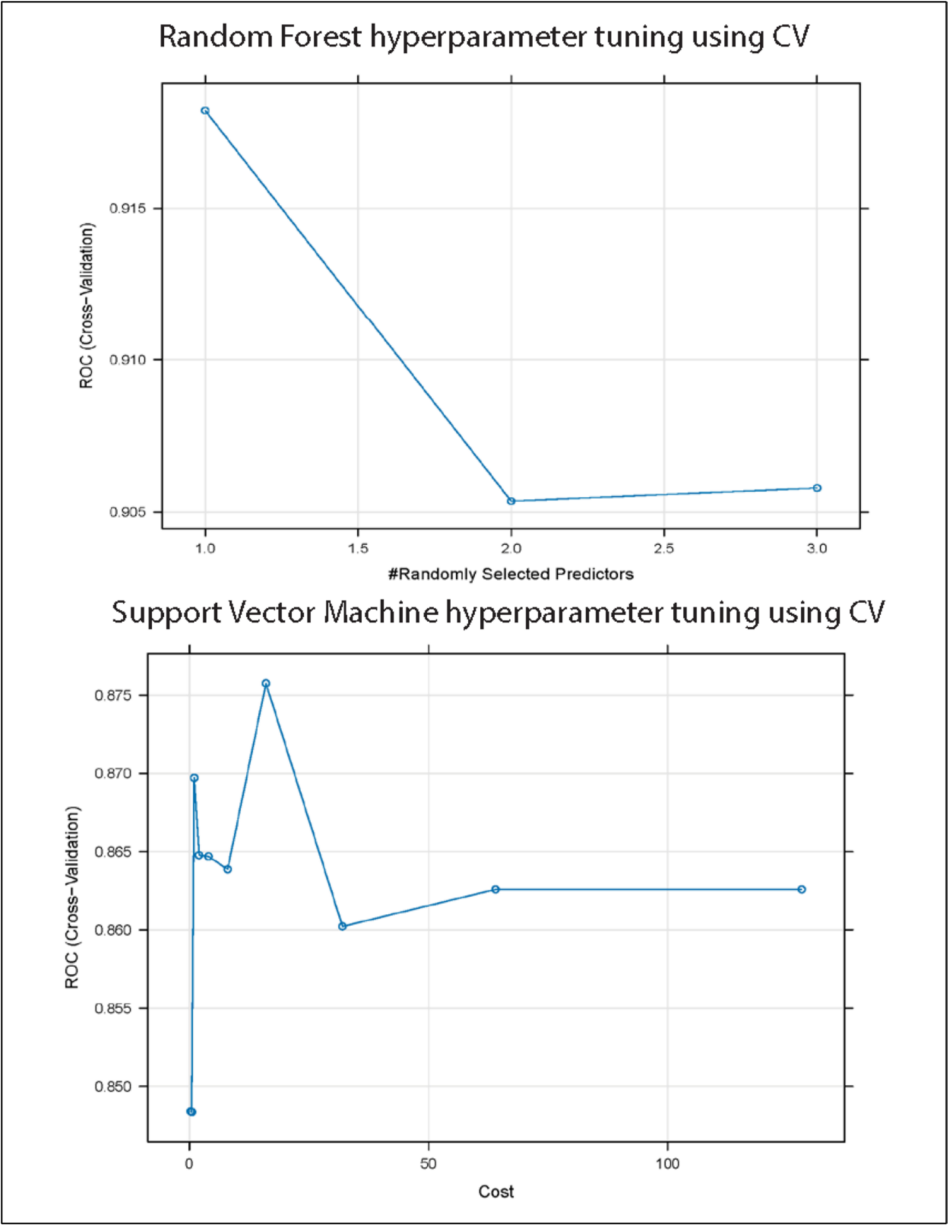
Cross-validated hyperparameter tuning of random forest and support vector machine models. Cross-validated ROC AUC is shown as a function of the Random Forest tuning parameter (mtry, top) and the Support Vector Machine cost parameter (C, bottom). Performance was estimated using tenfold-cross-validation on the training set

**Fig. 6:**
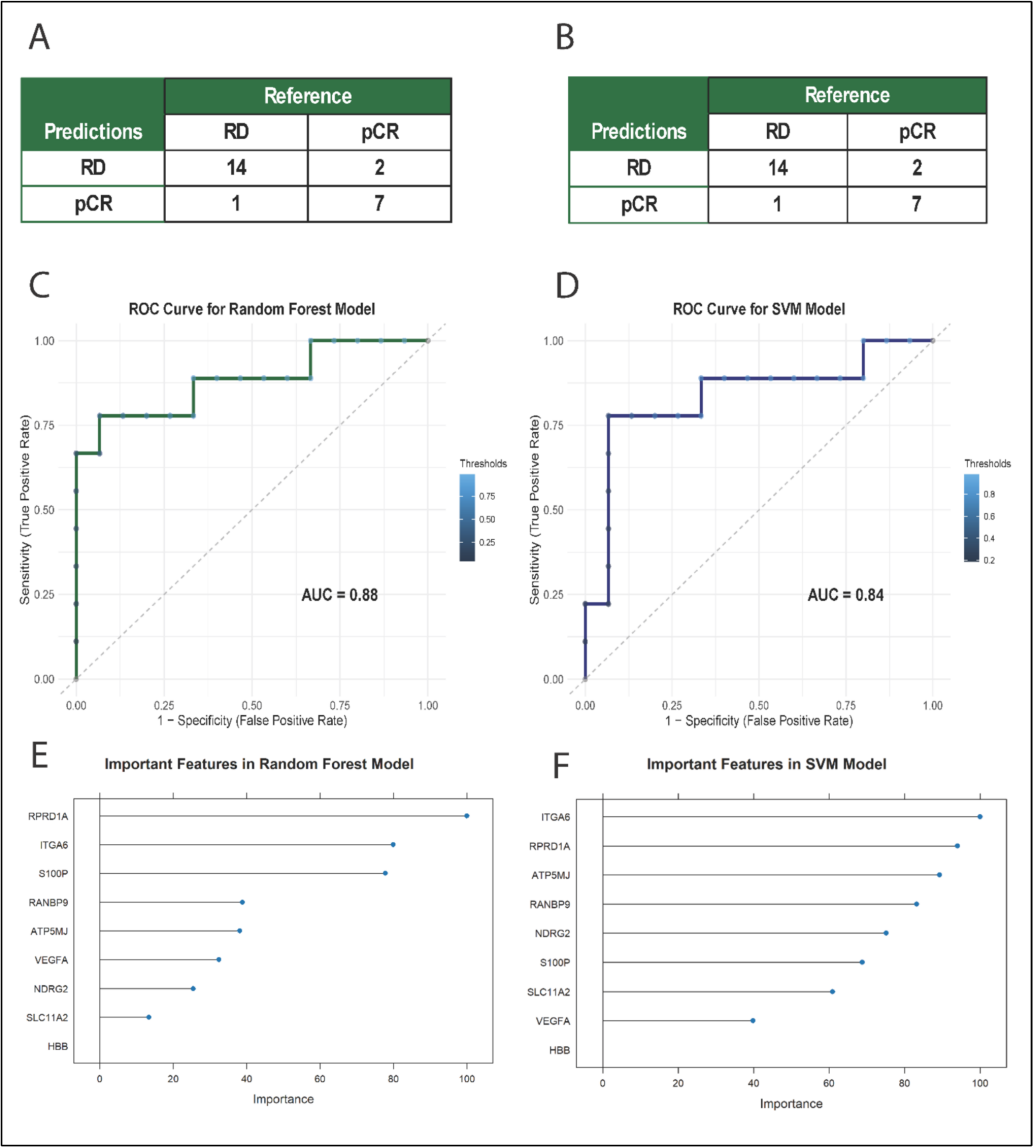
Evaluation of models’ performance and feature importance. (**A**) and (**B**) represent confusion matrices for validation sets for Random Forest (RF) and Support Vector Machine (SVM) models. (**C**) and (**D**) represent ROC curves for the validation set, comparing the performance of RF and SVM models. (**E**) and (**F**) represent feature importance rankings derived from RF and SVM models, showing key predictors influencing classification

Both models demonstrated discriminatory ability on the validation set (Fig. 5, Table 3). The RF model achieved a higher ROC AUC than the SVM model (0.88 vs 0.83), while sensitivity and specificity were comparable between the two models. ROC curves for both models are shown in Fig. 6, illustrating their performance on unseen data Table 4 (Fig. 6).

ROC analysis confirmed strong classification power, with the RF model yielding an AUC of 0.88 (Fig. 6C) and the SVM achieving 0.83 (Fig. 6D) on the validation set. Feature importance analysis from the RF model, based on LASSO-Boruta-selected genes, highlighted *RPRD1A, ITGA6, S100P, ATP5MJ, RANBP9, VEGFA, NRDG2,* and *SLC11A2* as top predictors (Fig. 6E). The SVM’s kernel-based analysis supported similar feature rankings (Fig. 6F).

### Biological significance of NAC gene signatures

**Fig. 7:**
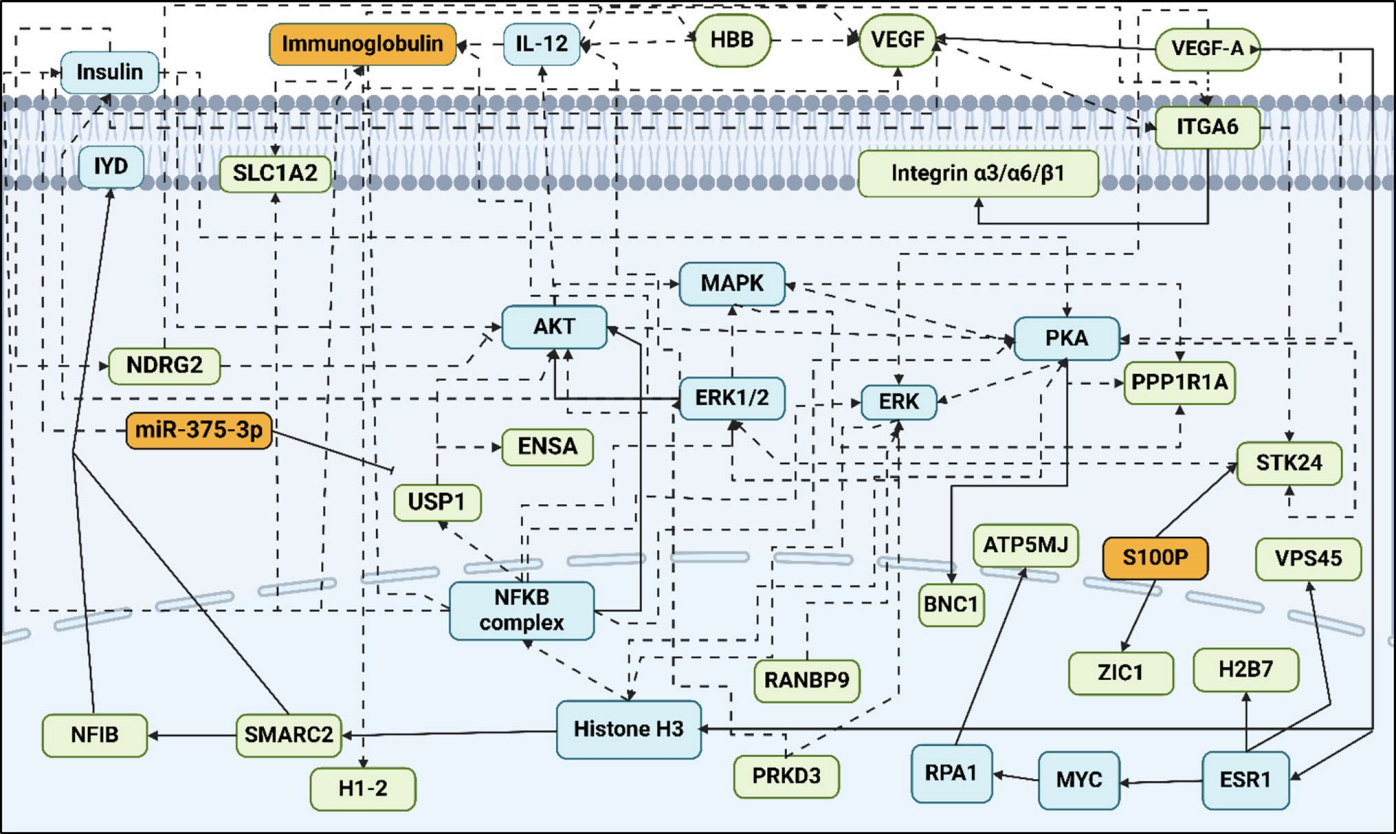
Schematic representation of the signaling pathways for the gene signatures predicting the response of triple-negative breast cancer patients to neoadjuvant chemotherapy. (green color—underexpression; orange color—overexpression; blue color—hub genes; dashed lines—indirect relationship; solid lines—direct relationship). Abbreviations: AKT, AKT serine/threonine kinase 1; ATP5MJ, ATP Synthase Membrane Subunit J; BNC1, Basonuclin Zinc Finger Protein 1; ERK, Extracellular Signal-Regulated Kinase; ERK1/2, Extracellular Signal-Regulated Kinase 1/2; ESR1, Estrogen Receptor Alpha Gene; ENSA, Endosulfine alpha; HBB, Hemoglobin Subunit Beta; H1-2, Linker Histone H1; H2B7, H2B clustered Histone 7; ITGA6, Integrin Subunit Alpha 6; IL-12, Interleukin 12 A; IYD, Iodotyrosine deiodinase; MAPK, Mitogen-activated protein kinase; miR-375-3p, MicroRNA 375-3p; MYC, Myc proto-oncogene; NFIB, Nuclear-Factor IB; NFKB, Nuclear-Factor Kappa B Subunit 1; NRDG2, Ny-my downstream-regulated gene 2; PKA, Protein Kinase A; PPP1R1A, Protein Phosphatase 1 Regulatory Inhibitor Subunit 1 A; PRKD3, Protein Kinase D3; RANBP9, RAN Binding Protein 9; RPA1, Replication protein A; SMARC2, SWI/SNF Related BAF Chromatin Remodeling Complex Subunit ATPase 2; SLC1A2, Solute Carrier Family 1 Member 2; STK24, Serine/Threonine Kinase 24; S100P, S100 Calcium-binding protein; VEGF, Vascular Endothelial Growth Factor; VEGFA, Vascular Endothelial Growth Factor A; VPS45, Vacuolar Protein Sorting 45 Homolog; USP1, Ubiquitin Specific Peptidase 1; ZIC1, Zinc Finger Protein of Cerebellum 1

To further explore the biological relevance of the 21 DEGs, we performed network analysis using IPA. The resulting interaction map (Fig. 7) revealed several hub genes including VEGF, VEGFA, and ITGA6 whose downregulation is closely associated with altered expression of surrounding genes in the network. Among them, ITGA6 emerged as the most interconnected node, linking to VEGFA, VEGF, Integrin alpha 3/6, USP1, and STK24. Its downregulation suggests a potentially disruptive effect on multiple downstream interactions. Similarly, PRKD3 showed strong regulatory links with AKT, NFKB, and ERK, while USP1 connected to ITGA6, AKT, and miR-375-3p, further underscoring their functional importance. Collectively, these hub genes may serve as regulatory anchors influencing TNBC chemo-responsiveness and represent potential targets for future therapeutic or biomarker development.

## Discussion

This study demonstrates the potential of machine-learning-based gene expression profiling as a predictive tool for neoadjuvant chemotherapy response in TNBC. Historically, clinical and histopathologic markers such as tumor grade, tumor-infiltrating lymphocytes, and Ki-67 proliferation index have been explored to predict NAC response [21–26]. However, our gene-expression-based machine-learning approach uniquely identifies molecular alterations directly associated with treatment outcomes, overcoming limitations of traditional predictors.

While expression profiling has widely predicted treatment responses in various cancers, similar studies specific to TNBC remain scarce [27–30]. Compared to previous machine-learning models, our approach demonstrated higher accuracy. Park and Yi (2022) previously reported an 86-gene signature achieving an AUROC of 89.1% in predicting NAC responses [31], while recent studies reported AUROCs of 71% and 80.2%, respectively [28, 29]. Our study successfully integrated multiple datasets, applied rigorous gene-selection methods, and achieved comparable or superior predictive performance.

### Key pathways in TNBC biology and therapy

KEGG and Ingenuity pathway analyses showed oxidative phosphorylation (OXPHOS), oxidative stress, diabetic cardiomyopathy, base excision repair (BER), mismatch repair (MMR), DNA synthesis, and cell-cycle regulation as significantly correlated with TNBC biology (Table 3), which have been previously linked to TNBC progression. TNBC cells undergo profound metabolic shifts. While many cancers favor aerobic glycolysis over mitochondrial OXPHOS [32], certain TNBC subsets paradoxically show enhanced OXPHOS activity[33]. For instance, the mitochondrial regulator LRPPRC is frequently overexpressed in TNBC and drives upregulation of electron transport chain components, increasing OXPHOS activity [33]. This metabolic flexibility can fuel aggressive growth but also generates reactive oxygen species (ROS) as byproducts of respiration. The NRF2 (NFE2L2) antioxidant response pathway, often activated via KEAP1 suppression, is a key survival mechanism; high NRF2 and low KEAP1 expression independently predict chemoresistance and poor survival in TNBC patients [34]. An integrative analysis confirms that oxidative stress and cellular senescence (OSCS) processes significantly impact TNBC progression [35]. Chronic oxidative stress can push cells toward senescence, a state of irreversible growth arrest, yet TNBC cells frequently circumvent full senescence, contributing to therapy resistance [35]. Pathway enrichment further identified “diabetic cardiomyopathy” among the top hits, likely reflecting shared metabolic stress features (e.g., hyperglycemia-like ROS overproduction and mitochondrial dysfunction) rather than literal cardiac pathology [36]. In sum, TNBC cells rely on reprogrammed energy metabolism and robust oxidative stress defenses, highlighting potential metabolic vulnerabilities.

TNBC is characterized by genomic instability and dysregulation of multiple DNA repair pathways. Homologous recombination (HR) repair deficiency is especially prevalent, as exemplified by the high frequency of BRCA1/2 mutations in TNBC (approximately 15–25% of patients) [37]. HR-deficient TNBC cells become reliant on backup repair mechanisms such as base excision repair (BER) to survive DNA damage [38]. Poly(ADP-ribose) polymerase 1 (PARP1), a key BER enzyme, is often hyperactivated in TNBC and represents a therapeutic target in HR-deficient tumors [38]. Notably, PARP1 functions at the crossroads of several repair pathways; it not only fixes single-strand breaks via BER but also contributes to nucleotide excision repair (NER), mismatch repair (MMR), and alternative end-joining[38]. Owing to this dependency, PARP inhibitors induce synthetic lethality in BRCA-mutated TNBC by crippling BER and trapping DNA repair complexes [38].

Mismatch repair defects, on the other hand, are rare in primary TNBC, and most TNBCs are microsatellite stable [39]. However, chemoresistance studies show that residual TNBC tumors can downregulate MMR gene expression, suggesting a dynamic loss of fidelity under therapeutic pressure [40]. Likewise, NER activity impacts treatment response: high expression of NER components such as ERCC1 (present in ~ 70% of TNBC tumors) is associated with poor response to platinum-based chemotherapy [41]. The pervasive DNA repair abnormalities in TNBC thus offer both challenges (e.g., therapy resistance) and opportunities (exploitable repair weaknesses via targeted therapies).

TNBC tumors are highly proliferative, reflecting widespread dysregulation of cell-cycle control. Most TNBC cases present with very high Ki-67 proliferation indices, indicating a large fraction of cells actively in S/G2-M phases[42]. Genomic analyses show that TP53, the guardian of the G1/S checkpoint, is mutated in > 80% of TNBCs[43]. Loss of p53 function, together with frequent alterations in other cell-cycle regulators (e.g., cyclin E/CDK2 upregulation or p16/RB pathways disruption), allows TNBC cells to bypass key checkpoints[43]. Consequently, DNA synthesis proceeds unchecked, leading to replication stress, an increased burden of DNA lesions, and a likely increase in the tumor’s reliance on DNA repair pathways. Some replication licensing factors (e.g., mini-chromosome maintenance helicases, proliferating cell nuclear antigen) are often elevated in TNBC, underlining heightened DNA synthesis [43, 44]. Targeting cell-cycle kinases (CDK1/2 and CDK4/6) is under investigation, though TNBC has shown variable sensitivity to such approaches [44]. Overall, the aggressive cell-cycle progression in TNBC underpins rapid tumor growth and influences the efficacy of cytotoxic treatments which typically target rapidly dividing cells.

Circadian rhythm perturbation emerges as an important yet underappreciated facet of TNBC biology [45]. The molecular clock, comprising transcriptional regulators such as CLOCK, BMAL1 (ARNTL), and PER/CRY proteins, normally coordinates cell proliferation, cell-cycle checkpoints, and DNA repair in a time-of-day-dependent manner [46]. In TNBC, this clock is frequently dysregulated. Transcriptomic analyses reveal that core clock genes PER2 and PER3 are significantly underexpressed in TNBC tumors (while PER1 is slightly elevated), and the clock output gene CRY2 is also reduced; conversely, certain clock factors (NPAS2, ARNTL2, NFIL3) are upregulated [47]. This shift indicates a loss of normal circadian control, which may contribute to unchecked cell division and impaired DNA damage responses.

Interestingly, circadian regulators intersect with hormone signaling pathways [46]. Although TNBC lacks estrogen receptor α expression by definition, the circadian protein CLOCK is paradoxically upregulated in ER-negative breast tumors[46]. Experimental evidence in ER-positive models shows that CLOCK can co-activate ERα-dependent transcription, and that another clock component, PER2, directly binds ER α to accelerate its proteasomal degradation [46]. Thus, in ER-positive breast cancer, the clock modulates estrogen signaling; conversely, in TNBC, the absence of ER α likely forces the clock network to interface with alternative growth pathways. Epidemiological studies link circadian disruption (e.g., chronic night-shift work) to increased breast cancer risk [46]. Preclinical research suggests that restoring circadian alignment or pharmacologically targeting clock components (for example, via the nocturnal hormone melatonin) can inhibit TNBC progression [48]. Therefore, TNBC tumors not only evade endocrine regulatory mechanisms but also show circadian clock disturbances, both of which likely fuel their aggressive, treatment-resistance phenotype.

### TNBC gene signatures identified by LASSO and BORUTA

In this study, we used LASSO and Boruta feature selection to derive an overlapping of 21 gene signatures that predict NAC response with ~ 90% accuracy in public TNBC datasets (validated via Random Forest and SVM classifiers). These particular genes, *S100P, RPRD1A, RANBP9, HBB, NDRG2, ITGA6, VEGFA, SLC11A2, ATP5MJ, STK24, HAT1, PDK3,* and *ZIC1*, were identified as most relevant predictors. This high-performance predictor suggests that the expression of these genes captures key biological determinants of chemosensitivity.

Interestingly, ITGA6 stands out as the most interconnected gene within the panel, linking to VEGFA, VEGF, Integrin alpha 3/6, USP1, and STK24. Integrin α6, a cell-surface receptor for extracellular matrix, is a known marker of basal stem-like tumor cells. High ITGA6 marks subpopulations with high tumor-initiating capacity and self-renewal [49]. Clinically, ITGA6 overexpression correlates with aggressive behavior; it is an independent adverse prognostic factor in ER-negative breast cancer and promotes tumor dissemination and metastasis [49]. These findings suggest that TNBC tumors enriched in ITGA6 are less likely to be eradicated by chemotherapy. Another key molecule implicated is VEGF-driven angiogenesis. VEGF signaling can maintain a chemo-resistant, stem-like niche [50]. Indeed, blocking VEGF–Neuropilin-2 binding in TNBC was recently shown to force cancer stem cells into differentiation, thereby sensitizing tumors to chemotherapy and limiting metastasis [50]. This highlights that an adequate vasculature and microenvironment, potentially reflected by our signature genes, modulate drug delivery and tumor cell survival under NAC. The convergence of ITGA6 and VEGF-related biology in our results supports the notion that therapies targeting the stem cell niche or tumor vasculature might improve NAC responses in TNBC.

Our signature also captures factors associated with epithelial-mesenchymal transition (EMT) and DNA damage tolerance, well-known drivers of chemoresistance. S100P, a calcium-binding protein, is frequently overexpressed in aggressive breast cancers and has been directly implicated in drug resistance [51]. Mechanistically, S100P binds and functionally inactivates p53, dampening p53-dependent apoptosis in response to DNA damage allowing cancer cells to survive chemotherapy and regrow, contributing to treatment failure [51]. S100P also signals via the receptor for advanced glycation end-products (RAGE) to activate pro-survival pathways (MAPK/ERK, PI3K/AKT, NFkB), promoting an EMT-like and invasive phenotype [51]. Likewise, the USP1 deubiquitinase, identified in our analysis, fosters EMT and chemoresistance [52]. USP1 in complex with WDR48 stabilizes critical TGF-β signaling components, thereby enhancing TGF-β-induced EMT and cell migration in TNBC. High USP1 activity has been linked to poor prognosis and therapy resistance in aggressive cancers, partly by stabilizing EMT transcription factors such as Snail which in turn promotes metastasis and resistance to platinum-based drugs likely due to a more mesenchymal, drug-tolerant state [52]. In contrast, STK24 (MST3) appears to act as a metastasis-suppressor kinase [53]. Strikingly, silencing STK24 was shown to increase breast cancer cell mobility, while overexpression curbed migration [53]. Loss of STK24 might thus remove a brake on EMT and invasion, aligning with a chemo-resistant phenotype [53]. Taken together, the presence of S100P, USP1, and potentially low STK24 in poor responders highlights the pivotal role of EMT and DNA damage response pathways in NAC resistance.

Recent studies confirm that NFIB, Nuclear-Factor I/B, is frequently overexpressed in TNBC, correlating with high-grade tumors, poor prognosis, and reduced chemotherapy response[54]. NFIB overactivity, often co-occurring with TP53 mutation, drives a pro-survival program: NFIB directly represses the CDKN1A/p21 gene, thus bypassing p53’s cell-cycle arrest and apoptotic functions [54]. Functionally, NFIB knockdown in TP53-mutant TNBC cells induces p21, leading to cell-cycle arrest and restored chemosensitivity to docetaxel [54]. This evidence aligns with our findings that NFIB is a top predictor of NAC response. Tumors with lower NFIB levels or intact p53/p21 signaling are more prone to chemotherapy-induced cell death, whereas NFIB-overexpressing tumors resist therapy by disabling p53-dependent checkpoints. On the other hand, ZIC1, zinc finger of the cerebellum 1, emerged as a putative tumor suppressor in our signature. ZIC1 is frequently downregulated in breast cancers often via promoter hypermethylation, and higher ZIC1 expression has been linked to most favorable prognosis [55]. In functional assays, ZIC1 re-expression curtails breast cancer growth by inactivating the pro-survival Akt/mTOR pathway and downregulating survivin BIRC5, a key anti-apoptotic protein often linked to therapy resistance [56]. In vivo, elevated ZIC1 levels suppress tumor formation and induce apoptosis via upregulation of mitochondrial and caspase pathways [56]. Thus, ZIC1 loss may enable tumor cells to evade chemotherapy-induced apoptosis, whereas ZIC1-positive tumors are more chemo-responsive. The inclusion of ZIC1 in our predictive panel suggests that assessing tumor-suppressive gene activity alongside oncogenic drivers improves identification of chemosensitive tumors.

In conclusion, this study applied multiple machine-learning approaches to derive gene signatures predictive of response to anthracycline-based neoadjuvant chemotherapy in TNBC. The resulting predictive model demonstrated robust performance and promised to serve as a foundation for clinical translation following further validation.

## Supplementary Information

Below is the link to the electronic supplementary material.

Supplementary file1 (PDF 627 KB)

## Data Availability

The datasets presented in this study can be found in online repositories. The names of the repository/repositories and accession number(s) can be found in the article.
