# Supplementary material for: Development and validation of a highly accurate multigene gene expression biomarker to predict chemotherapy response in primary triple-negative breast cancer
Source: Breast Cancer Res Treat. 2026 Mar 26;217(1):1. doi: 10.1007/s10549-026-07950-4 (PMC13021716; doi:10.1007/s10549-026-07950-4)
Supplement: Supplementary file 1 — Supplementary file1 (PDF 627 KB) [file 10549_2026_7950_MOESM1_ESM.pdf]

Article title: Development and validation of a highly accurate Multigene Gene expression biomarker to predict chemotherapy response in primary triple-negative breast cancer

Journal name: Breast Cancer Research and Treatment

Author names: **Soukaina Amniouel[1], Mohsin Saleet Jafri[1,2]**

Affiliation:

[1]School of Systems Biology, George Mason University, Fairfax, VA 22030, USA
[2]Center for Biomedical Engineering and Technology, University of Maryland School of Medicine, Baltimore MD 21201, USA

*** Correspondence:**
Corresponding Author
sjafri@gmu.edu

Online Resource 1: Combined visualization of Normalization and Principal Component Analysis (PCA) of TNBC data. (A) Boxplot of normalized data across samples, demonstrating the distribution of values after normalization. (B) PCA analysis of normalized data, revealing sample clustering.

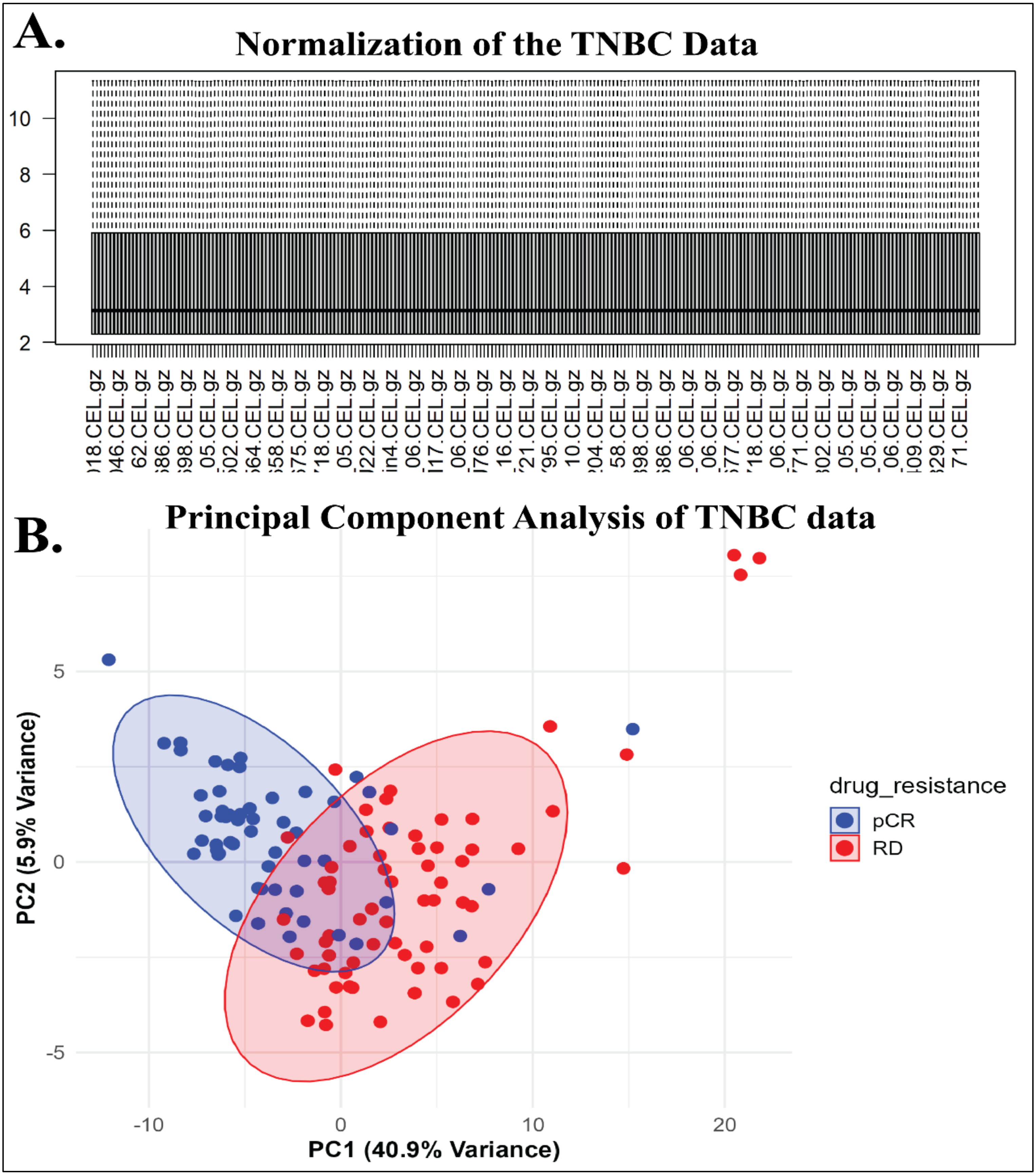

<u>Online Resource 1</u>: Combined visualization of Normalization and Principal Component Analysis (PCA) of TNBC data. (A) Boxplot of normalized data across samples, demonstrating the distribution of values after normalization. (B) PCA analysis of normalized data, revealing sample clustering.

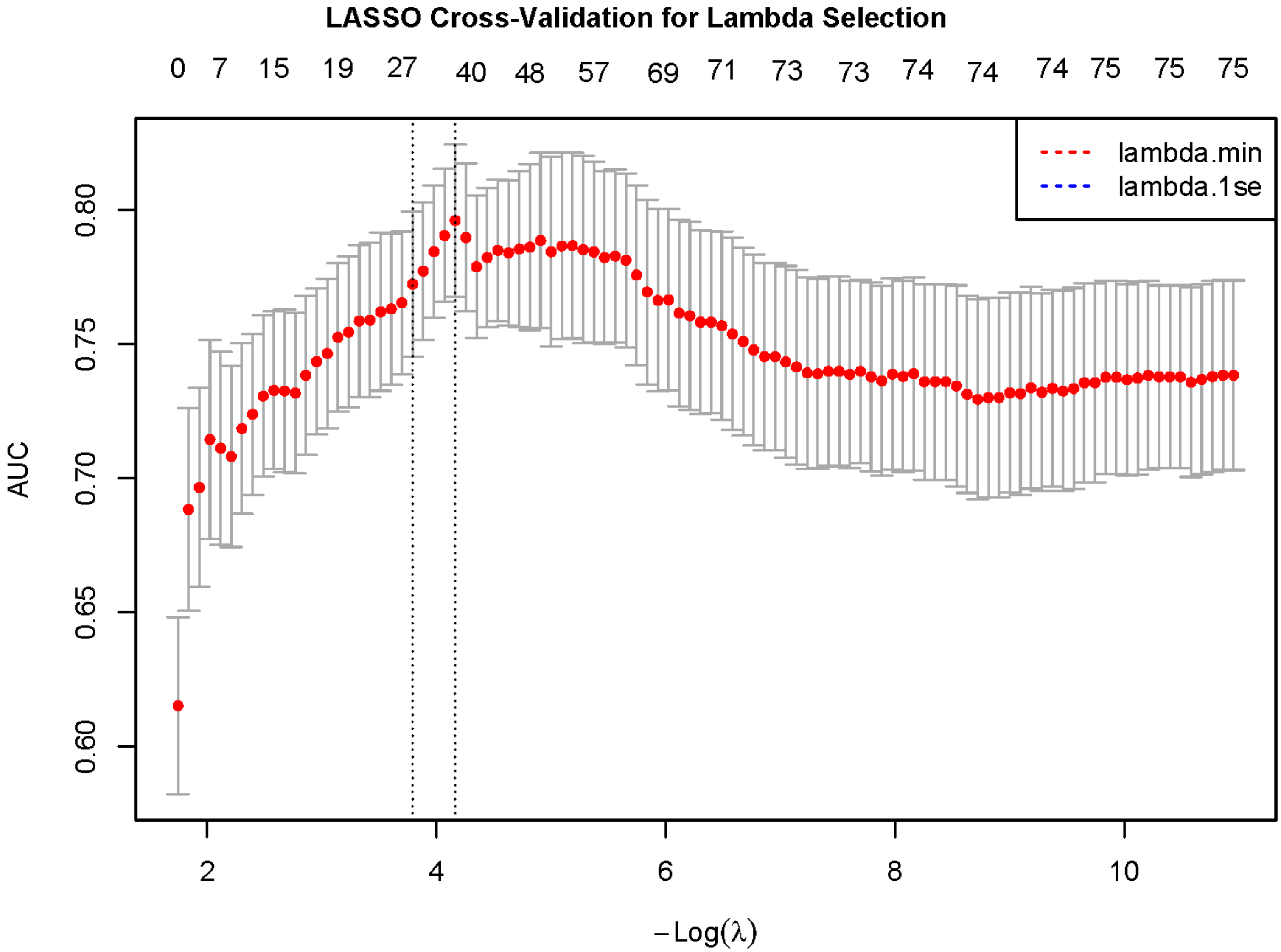

<u>Online Resource 2</u>: Cross-validation curve for LASSO regularization parameter selection. The mean cross-validated performance across candidate values of the regularization parameter (λ) is shown, with error bars indicating ±1 standard error. The dashed vertical line denotes the selected λ based on the one-standard-error rule. Cross-validation was performed using 10 folds on the training set.

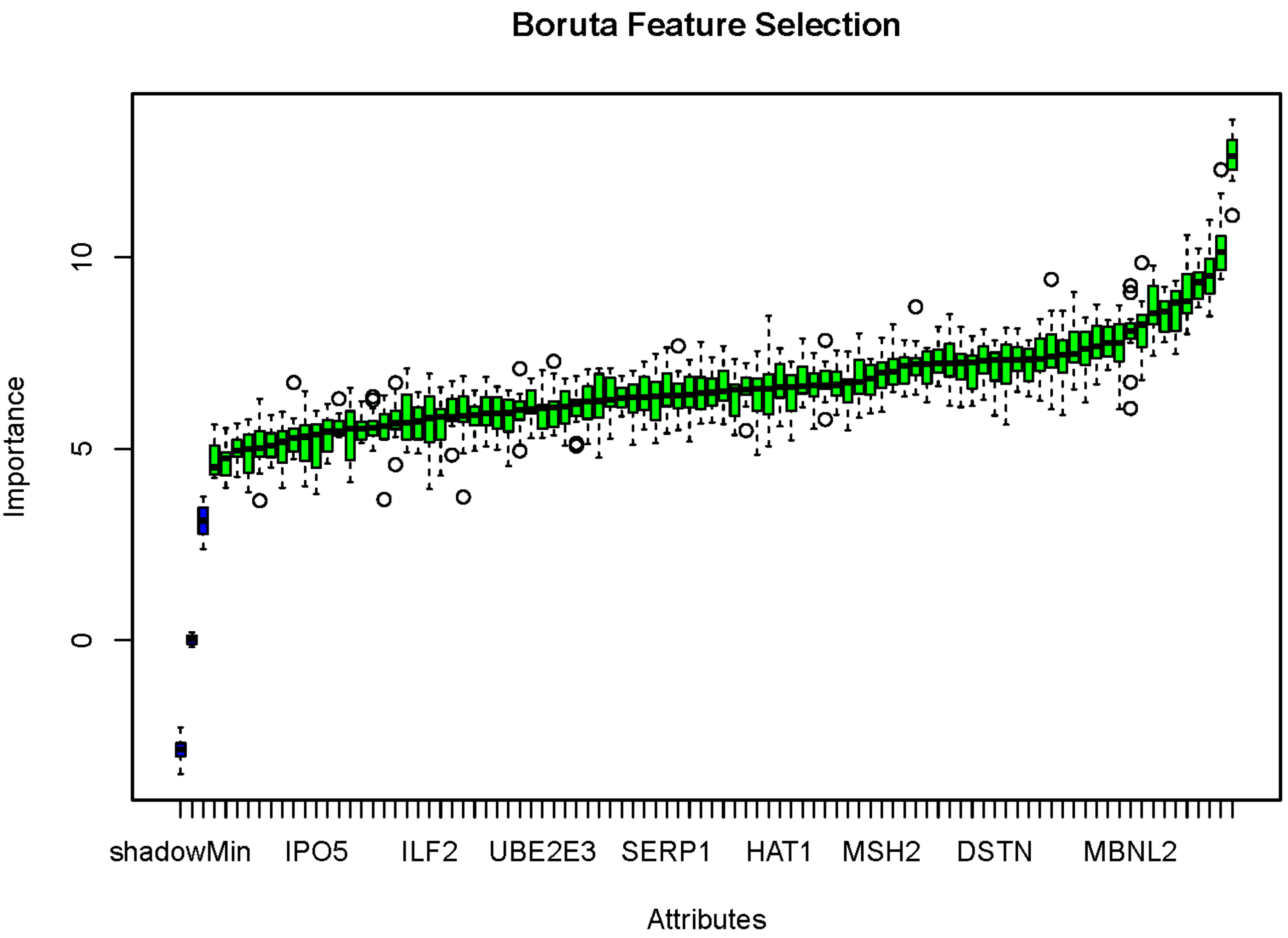

<u>Online Resource 3:</u> Boruta feature selection based on Random Forest importance. Features were evaluated using the Boruta algorithm on the training set. Confirmed features are shown based on their mean importance across Random Forest iterations, while rejected and tentative features are excluded. This analysis identifies features that are significantly more informative than randomized shadow features.

A

mtry	ROC	Sens	Spec
1	0.9182104	0.9340659	0.7107143
2	0.9053571	0.9269231	0.6982143
3	0.9057889	0.9049451	0.6982143

B

C	ROC	Sens	Spec
0.25	0.8484105	0.8626374	0.6803571
0.50	0.8483713	0.8769231	0.6946429
1.00	0.8697214	0.8912088	0.6803571
2.00	0.8647763	0.8983516	0.6428571
4.00	0.8646880	0.8989011	0.6696429
8.00	0.8638834	0.8917582	0.7089286
16.00	0.8757555	0.8917582	0.6946429
32.00	0.8602237	0.8917582	0.7089286
64.00	0.8625883	0.8851648	0.7232143
128.00	0.8625883	0.8851648	0.7232143

Online Resource 4: Hyperparameter tuning of Random Forest and Support Vector Machine models using cross-validation. (A) Cross-validated model performance for the Random Forest classifier across different values of the *mtry* parameter, shown in terms of ROC AUC, sensitivity, and specificity. **(B)** Cross-validated model performance for the Support Vector Machine classifier across candidate values of the cost parameter (*C*), shown in terms of ROC AUC, sensitivity, and specificity. All performance metrics were estimated using 10-fold cross-validation on the training set.
